# Leptospirosis in Rio Grande do Sul, Brazil: An Ecosystem Approach in the Animal-Human Interface

**DOI:** 10.1371/journal.pntd.0004095

**Published:** 2015-11-12

**Authors:** Maria Cristina Schneider, Patricia Najera, Martha M. Pereira, Gustavo Machado, Celso B. dos Anjos, Rogério O. Rodrigues, Gabriela M. Cavagni, Claudia Muñoz-Zanzi, Luis G. Corbellini, Mariana Leone, Daniel F. Buss, Sylvain Aldighieri, Marcos A. Espinal

**Affiliations:** 1 Department of Communicable Diseases and Health Analysis, Pan American Health Organization, Washington, D.C., United States of America; 2 Laboratório de Referência Nacional para Leptospirose, Centro Colaborador da Organização Mundial da Saúde, Fundação Oswaldo Cruz, Rio de Janeiro, Brazil; 3 Faculdade de Medicina Veterinária, Departamento de Medicina Veterinária Preventiva, Laboratório de Epidemiologia Veterinária, Universidade Federal do Rio Grande do Sul, Porto Alegre, Rio Grande do Sul, Brazil; 4 Secretaria de Saúde do Rio Grande do Sul, Porto Alegre, Rio Grande do Sul, Brazil; 5 Instituto de Pesquisas Veterinárias Desidério Finamor (IPVDF), Fundacão Estadual de Pesquisa Agropecuária (FEPAGRO), Eldorado do Sul, Rio Grande do Sul, Brazil; 6 Secretaria da Agricultura Pecuária do Sul, Porto Alegre, Rio Grande do Sul, Brazil; 7 Division of Epidemiology and Community Health, School of Public Health, University of Minnesota, Minneapolis, Minnesota, United States of America; 8 Laboratório de Avaliação e Promoção da Saúde Ambiental, Instituto Oswaldo Cruz, Fundação Oswaldo Cruz, Rio de Janeiro, Brazil; University of California, San Diego School of Medicine, UNITED STATES

## Abstract

**Background:**

Leptospirosis is an epidemic-prone neglected disease that affects humans and animals, mostly in vulnerable populations. The One Health approach is a recommended strategy to identify drivers of the disease and plan for its prevention and control. In that context, the aim of this study was to analyze the distribution of human cases of leptospirosis in the State of Rio Grande do Sul, Brazil, and to explore possible drivers. Additionally, it sought to provide further evidence to support interventions and to identify hypotheses for new research at the human-animal-ecosystem interface.

**Methodology and findings:**

The risk for human infection was described in relation to environmental, socioeconomic, and livestock variables. This ecological study used aggregated data by municipality (all 496). Data were extracted from secondary, publicly available sources. Thematic maps were constructed and univariate analysis performed for all variables. Negative binomial regression was used for multivariable statistical analysis of leptospirosis cases. An annual average of 428 human cases of leptospirosis was reported in the state from 2008 to 2012. The cumulative incidence in rural populations was eight times higher than in urban populations. Variables significantly associated with leptospirosis cases in the final model were: Parana/Paraiba ecoregion (RR: 2.25; CI_95%_: 2.03–2.49); Neossolo Litolítico soil (RR: 1.93; CI_95%_: 1.26–2.96); and, to a lesser extent, the production of tobacco (RR: 1.10; CI_95%_: 1.09–1.11) and rice (RR: 1.003; CI_95%_: 1.002–1.04).

**Conclusion:**

Urban cases were concentrated in the capital and rural cases in a specific ecoregion. The major drivers identified in this study were related to environmental and production processes that are permanent features of the state. This study contributes to the basic knowledge on leptospirosis distribution and drivers in the state and encourages a comprehensive approach to address the disease in the animal-human-ecosystem interface.

## Author Summary

Leptospirosis is a bacterial disease affecting humans and several animal species, which serve as reservoirs for infection. Contamination happens through exposure to urine of infected animals in water and soil.

A better understanding of the factors that affect the transmission of the disease, incorporating the relationship between humans, animals, and ecosystems within the One Health approach, would allow for tailored prevention and control measures at the local level. The aims of this study were to analyze the distribution of leptospirosis human cases and the possible factors that influence the transmission of the disease in the state of Rio Grande do Sul, as well as provide evidence to support the development of interventions and guide new studies in the region.

Leptospirosis cases and possible environmental, socioeconomic, and livestock factors were analyzed. The study used data by municipality (all 496) obtained via an open access database. Georeferenced maps were constructed and statistical analysis performed for 26 variables. A multivariable regression analysis was carried out.

An annual average of 428 human cases of leptospirosis was reported in the state from 2008 to 2012. The cumulative incidence in rural populations was eight times higher than in urban populations. Variables significantly associated with leptospirosis cases in the final model were: ecoregion, type of soil and to a lesser extent tobacco and rice production.

Results showed that leptospirosis cases were concentrated in the state capital and in specific ecoregions. Environmental and agricultural production processes were identified as possible risk factors and these conditions are probably permanent characteristics of the state. This study contributes to the knowledge on leptospirosis distribution and risk factors in the state, and also highlights the importance of a holistic view and intersectoral approach in preventing the disease.

## Introduction

Even though leptospirosis is now recognized as a disease of epidemic potential with a significant health impact in many parts of the world, it remains a neglected disease. Its burden is estimated at 500,000 severe human cases per year worldwide, but a WHO expert group recently put its annual global incidence at 1.03 million people with 58,900 deaths [1,2]. Nevertheless, leptospirosis continues to be a silent disease [3], mainly due to the paucity of data in many countries, including of the Americas [4,5].

The *Leptospira* bacteria may also affect a wide variety of animal species, both wild and domestic, which serve as reservoirs for human infection [6]. The diversity of animal carriers represents a significant challenge for prevention and control [7]. Exposure to water and soil contaminated by the urine of infected animals is the most common route of transmission to people and domestic animals [6].

Leptospirosis is an excellent example for the “One Health” approach, where the relationship between humans, animals and ecosystems is studied to improve knowledge on a disease and to enhance collaborative intersectoral and multidisciplinary control strategies [8]. The One Health working definition states that “it is feasible to integrate human, animal, and environmental health efforts to predict and control certain diseases at the human–animal–ecosystem interface; integrated approaches that consider human, animal, and environmental health components can improve prediction and control of certain diseases” [9]. Yet this approach is rarely used to advance knowledge on leptospirosis transmission, develop evidence-based policies, and create tools to save human lives and reduce the impact on domestic animals.

Leptospirosis is one of the major neglected diseases in Latin America [10]. It has been reported in a variety of settings, from large urban centers, to remote rural areas [11,12,13,14,15]. Socioeconomic drivers include living in dense urban or peri-urban areas with inadequate waste collection and sanitation, which is often associated with vulnerable populations [16]. Leptospirosis has been linked to poverty, lack of water and sanitation, and poor housing conditions [12,17].

Environmental drivers have been identified in previous studies. Heavy rains or floods have been linked to a higher number of cases of leptospirosis [12,16,18,19,20,21]. Alkaline and neutral soil are suspected of promoting a longer survival of the bacteria, especially in young, not-yet-structured soils like those of volcanic origin [12,22]. In addition, soil temperature and proximity to water bodies were also reported as potential enablers for bacterial survival [23]. Leptospirosis is also considered an occupational disease, affecting rice laborers, sewer workers, animal handlers and gold miners [6,24,25].

A better understanding of the drivers for leptospirosis would provide crucial information for decision-makers to be able to target risk areas for priority interventions. Indeed, the current gaps in scientific and technological knowledge hinder the detection of cases and limit surveillance and control programs. Finally, because the instruments needed for control and elimination–such as broad rapid tests and vaccines–are not available at this time, leptospirosis is not considered to be “tool ready” and as a result, it is not targeted through large-scale global initiatives.

Brazil is the fifth most populous country in the world (approximately 200 million people) and has the seventh highest gross domestic product (USD 2,250,673). Even though the economy has been growing steadily, there are still around 20 million people (10% of the population) living in poverty in the country [26]. This population group is the most vulnerable to neglected diseases and other poverty-related infections. Previous studies have demonstrated the impact of neglected tropical diseases in Brazil and the need to develop new tools and technologies to fight them [27,28].

There is no accurate surveillance system in place for leptospirosis globally or in the Americas; however, some countries’ surveillance systems include the disease and estimates of its public health burden are available [5,29]. Notification of human leptospirosis is mandatory in Brazil and an annual average of 3,888 cases with 9.48% fatality are officially reported by the country’s surveillance system [30].

The Brazilian state of Rio Grande do Sul ranks fifth in the incidence rate (4.7 cases per 10,000 population) and presents around 15% of the total number of cases in the country [31]. In a previous study conducted to identify high transmission areas and possible ecological components of leptospirosis transmission in Rio Grande do Sul, the highest incidence rates were found in the coastal sedimentary areas with low altitude and predominantly agricultural land use in the central valley [32]. The state economy is based on agribusiness, including cattle and rice paddies, with an associated increased risk of leptospirosis in some areas that needs to be evaluated.

The objective of this study is to analyze the distribution of human cases of leptospirosis in the state of Rio Grande do Sul and explore possible drivers using the One Health approach. This analysis may orient further studies of the disease in the human-animal-ecosystem interface and the results of this study may be used as evidence to inform decision makers in the state.

## Methods

### Study design and data collection

An ecological study was carried out using aggregated data by municipality to analyze the situation of leptospirosis in all 496 municipalities (corresponding to the second subnational administrative level) in the state of Rio Grande do Sul, Brazil, between 2008 and 2012.

A geo-coded database was created using different sources. Variables were either downloaded or created from original sources. The data source used for each variable is described in the S1 Supporting Information.

Human leptospirosis case data, de-identified and aggregated at the municipality level, were obtained from the Ministry of Health of Brazil national surveillance system database (acronym in Portuguese SINAN) [33]. All case data were publicly available by open consultation on the government website.

Seven environmental variables (ecoregion, type of soil, temperature, precipitation of the wettest month, altitude, slope of the land (hill incline), and drainage) were gathered from diverse “open-access” data sources and used in the study. When disaggregated, the ecoregion and type of soil variables turned into six and twelve variables, respectively. Altitude and hydrology information used in the background were obtained from the USGS-EROS, HYDRO1k Elevation Derivative Database [34]. Bioclimatic variables were calculated from monthly temperatures and rainfall values [35]. Geo-processing techniques were applied to assign and measure environmental variables for each municipality. Data for the socioeconomic variables (gross domestic product per capita, Gini coefficient, illiteracy rate) were gathered from the Brazilian Institute of Geography and Statistics (acronym in Portuguese IBGE) [36]. As the economy of Rio Grande do Sul is based on agribusiness, variables related to rice and tobacco production were collected from the IBGE database and data on bovine raising were provided by the Department of Agriculture of the state of Rio Grande do Sul and the Federal University of Rio Grande do Sul [37].

### Definitions

Leptospirosis cases: According to the Ministry of Health of Brazil, human cases of leptospirosis are those presenting clinical symptoms consistent with the disease and confirmed by laboratory diagnosis either with ELISA–IgM or MAT [38]. These laboratory confirmation techniques are available at the state level at the Central Public Health Laboratory, which is part of the National Public Health Laboratory Network. In Brazil, a case could also be confirmed by clinical-epidemiological criteria (selected symptoms with epidemiological history) [39]. All cases in the SINAN database were considered confirmed. In the original database, the cases were classified according to area of residence (urban, peri-urban or rural) [39].

Cumulative incidence: the number or proportion of a group (cohort) of people who experience the onset of a health-related event during a specific time interval [40]. In this study, it was estimated per 10,000 inhabitants.

Criteria for risk stratification: The criteria used were based on a previous risk stratification study conducted in Nicaragua [12]. In summary, geographical areas were classified into two categories: i) Silent Area (no cases were reported during the study period) and ii) Productive Area (active transmission was reported during the study period) that could be an endemic or critical area (higher quintile).

### Study area

Rio Grande do Sul is the southernmost state in Brazil bordering Argentina and Uruguay. The state covers an area of 281,731,445 km², divided into 496 municipalities. In 2010, the population was 10,693,929 inhabitants, 85.1% of which lived in urban areas, among them the Porto Alegre metropolitan area where 15% of the state population resides (1,472,482 inhabitants) [41].

In 2010, the state had the 4^th^ highest gross domestic product per capita in the country and the Gini index (0.5472) was lower than the national level [36]. There are three major economic regions: 1) the south with greater land concentration, large cattle raising farms, and mechanized plantation of rice, soybean and wheat. This area also presents higher income inequality; 2) the northeast region, which includes the state capital, with more industries and predominantly small properties; and 3) the northern region, mostly colonized by European immigrants, with higher forest coverage, valleys, and plain areas with small agricultural lands. The most common agrarian structure in the state (90% of properties) is a small family farm covering an area smaller than 100 hectares [42].

The hydrology of the state is basically divided into two main areas: the La Plata and Atlantic East Coast watersheds. The La Plata watershed is located in the northern area bordering with Argentina, and comprises the Uruguay River and mayor tributaries (Caboa, Pelotas, Ibicui and Mirinay). The Atlantic East Coast watershed is mainly shaped by the Guaiba and Litoranea basins. The Guaiba basin includes tributaries of the Dos Patos coastal lagoon, the Jacui and Tacuari rivers, which run in the central area of the state and wash its most populated areas; other tributaries include the Sinos and Cai rivers, which also flow into the Dos Patos lagoon. (S2 Supporting Information). The Litoranea basin encloses the Camaqua and Piratini rivers also flowing into the lagoon. The city of Porto Alegre is located in the Atlantic East Coast watershed.

### Data analysis

Two types of analyses were carried out to investigate associations between the risk of human leptospirosis and 26 variables selected as possible environmental, socioeconomic or livestock drivers: 1) a spatial analysis and thematic mapping of possible drivers, showing the municipalities in the higher quintiles of rates over the distribution of the variable also divided by quintiles (range cuts) and 2) a statistical analysis with univariate and multivariable regressions as described below.

The spatial analysis consisted in computing zonal statistics and surface for the environmental variables, in addition to the geocoding of health and socioeconomic data. ArcGIS zonal statistics by municipality (min, mean, max, standard deviation, range) were calculated for the altitude, slope, temperature, and rain variables. Geo-processing geometric intersection of environmental features shaped the municipal surface of ecoregions and soils. Quintile thematic mapping was conducted once the municipal statistics of environmental, health and socioeconomic variables were geo-processed.

For the multivariable regression, the dependent variable was the case count per municipality and the independent variables all 26 possible drivers. The ecoregion and soil variables were dichotomized as follows: they were coded 0 when they were a minority (when land coverage or proportion was less than or equal to 50% of the municipality) and they were coded 1 when they represented a majority (when land coverage or proportion was above 50.01% of the municipality.) The proportion of farms with up to ten animals per farm (small farms) was dichotomized in the same fashion. In addition, two bovine-related variables were created and used as continuous variable: the proportion of farms per km^2^ and the proportion of bovines per km^2^. Productive processes such as the cultivation of tobacco and rice were analyzed on a unit of 10,000 tons.

Predictors were analyzed with negative binomial regression (NB) and robust variance was used to estimate the relative risk (RR) and 95% confidence interval (CI) of the estimates [43]. The link function used was the default logit-function and the offset was the natural log-transformed number of population per municipality.

Univariate analyses were first run for each of all 26 variables and 14 were preselected due to P ≤ 0.15. Variance inflation factors (VIF) were estimated to verify the relations among all selected independent variables and check for potential collinearity. When a high VIF was found (VIF>4), the variable with a lower P-value was eliminated and the process was reiterated until only variables with a VIF<4 were left. Confounding effects were investigated by checking changes in the point estimates of the variables that remained in the model. Parameters with changes in their estimates > 25% were considered confounders and were retained during the variable selection process. Finally, two-way interaction terms between environmental variables with biological plausibility were investigated (slope and altitude, altitude and drainage, altitude and precipitation of the wettest month (mm)). We used deviance performance as a goodness of fit test for the overall model. Results of the variables with P value > 0.15 are included in the S3 Supporting Information.

Statistical tests were performed for over-dispersion evaluation and model fit comparison using literature-recommended approaches [44]. For the negative binomial model, the dispersion parameters were tested for difference with chi-squared statistics. To compare goodness of fit between pairs of the proposed regression models, Vuong statistics were calculated [45]. The following models were compared: Poisson regression, zero-inflated negative binomial (ZINB) and a negative binomial regression (NB) [46]. The differences in AIC and Vuong statistics were computed for all pairs of non-nested models (i.e., Poisson vs. ZINB, Poisson vs. NB, NB vs. ZINB). Based on the AIC and Vuong tests, the negative binomial regression model fit the data better in comparison with others. More details about the procedures carried out in order to compare models can be found in the S4 Supporting Information.

## Results

### Cases, cumulative incidence and risk stratification

During the study period, 2,141 confirmed cases of leptospirosis were reported with an average of 428 cases annually. The yearly incidence remained similar over the period (412–543 cases), except for a lower than average number of cases (277) in 2012 (Table 1).

**Table 1:** Human cases of leptospirosis by area of residence, Rio Grande do Sul, 2008–2012.

Year	Urban	%	Periurban	%	Rural	%	Unknown	%	Total	% *
**2008**	129	31%	16	4%	175	42%	92	22%	412	19%
**2009**	129	28%	20	4%	214	47%	92	20%	455	21%
**2010**	137	30%	15	3%	229	50%	73	16%	454	21%
**2011**	181	33%	18	3%	249	46%	95	17%	543	25%
**2012**	101	36%	17	6%	114	41%	45	16%	277	13%
**Total**	7	32%	86	4%	981	46%	397	19%	2141	100%

*Relative to the total during the 5 years

Source: SINAM, Ministry of Health of Brazil.

A total of 233 municipalities out of 496 reported at least one case (range 1–208) during the study period. Among the 263 municipalities not reporting cases, most had small populations (less than 10,000 people). On average, there were 4.32 cases per municipality.

**Fig 1:**
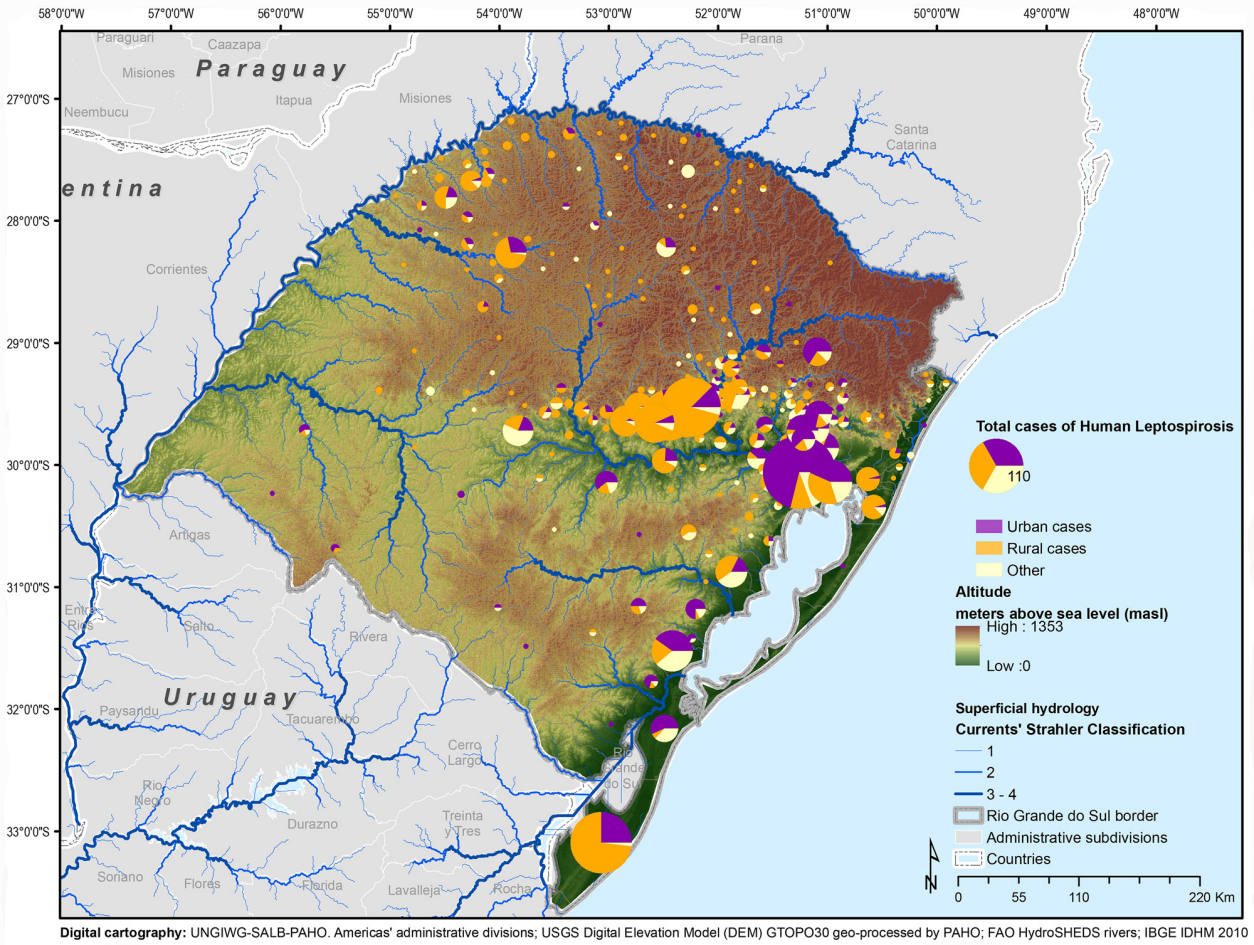
Human cases of leptospirosis according to the residence site, by municipality, Rio Grande do Sul, 2008–2012.

A high number of urban cases were clustered in the Metropolitan Region of Porto Alegre (50.52% of all urban cases) and groups of predominantly rural cases were located in the Center Oriental Region along the *Jacui* and *Tacuari* rivers and in the Southeast Region, near the great lagoons (Fig 1). Forty-seven municipalities concentrate 79.40% of the cases. Most of these municipalities are located in the *Atlantic East Coast* watershed, specifically in the *Jacui-Tacuari* and *Cai* basins (1,116 cases).

**Fig 2:**
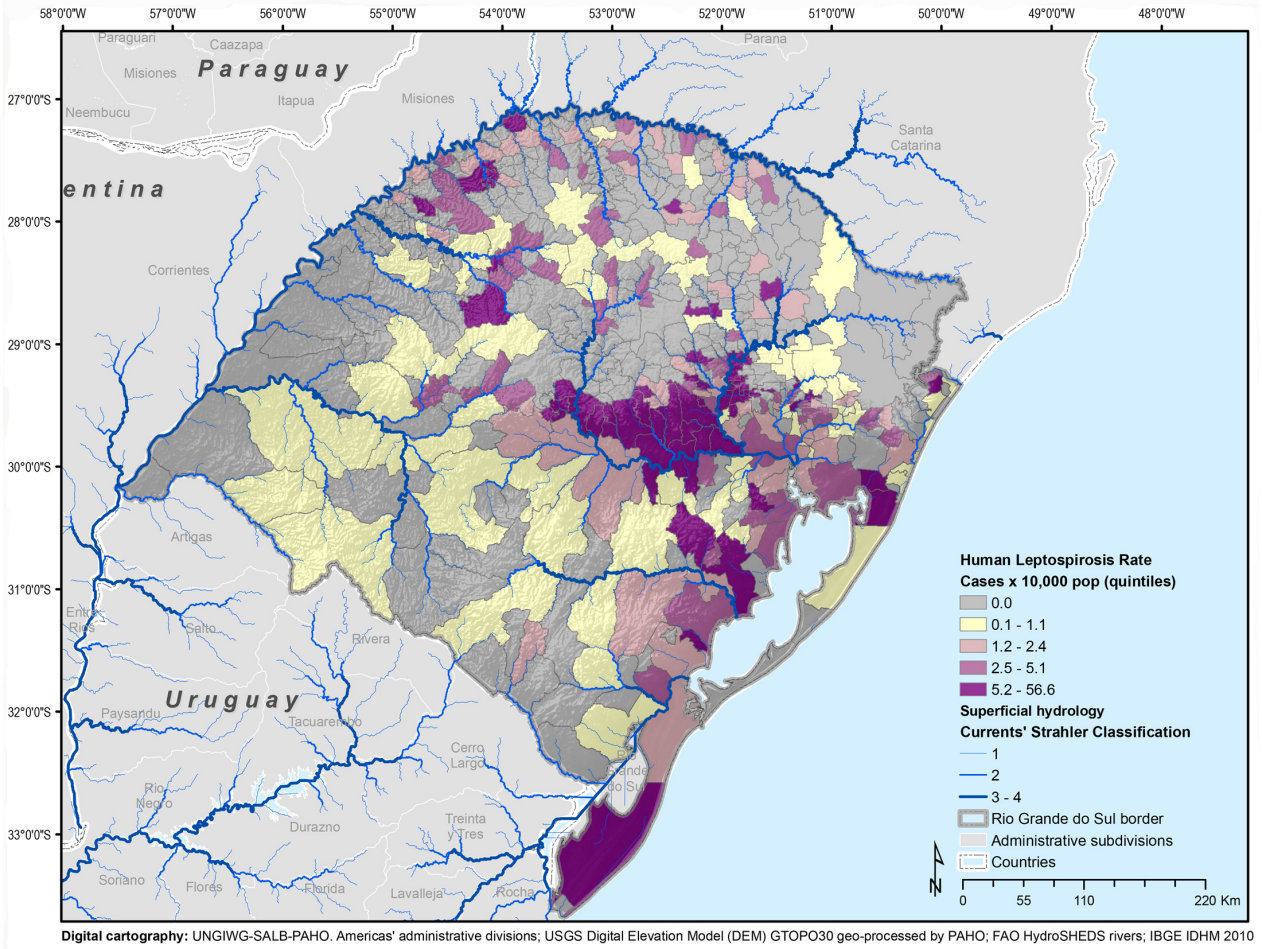
Cumulative incidence of leptospirosis (10,000 habitants), by municipality, Rio Grande do Sul, 2008–2012.

The cumulative incidence for the entire state was 2 cases per 10,000 inhabitants (range: 0–56.56). The spatial distribution of the municipalities’ cumulative incidence by quintiles, as well as of those not reporting cases, are presented in Fig 2. The cumulative incidence for rural areas (6.16 per 10,000 people) was eight times higher than for urban areas (0.74 per 10,000 people).

Risk stratification was carried out for the 496 municipalities in Rio Grande do Sul to inform planning of leptospirosis prevention and control activities. It shows that out of the 496 municipalities in the state, 263 (53.02%) can be considered silent areas. Among the 233 productive areas (46.98%), 58 were found to be endemic and 75 municipalities were considered critical areas (S5 Supporting Information). These 75 municipalities reported 1,766 cases (82.48%) out of the total of 2,141 cases in the entire state.

### Exploratory analysis of possible drivers

#### Environmental variables

**Fig 3:**
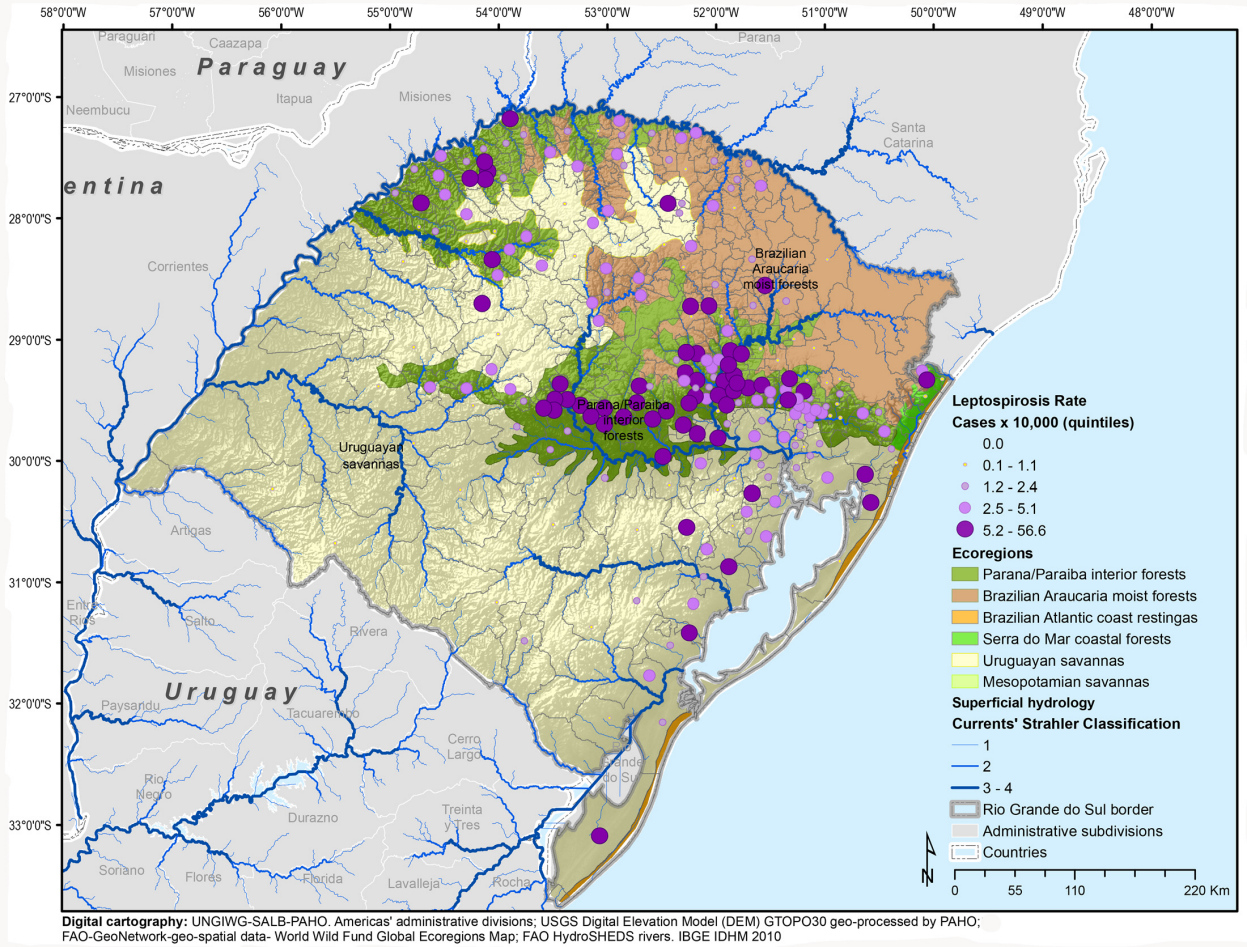
Cumulative incidence for leptospirosis and ecoregions, Rio Grande do Sul, 2008–2012.

The state of Rio Grande do Sul has six ecoregions. The largest extension is the Uruguayan savanna, covering 230,291.4 km² or 64.41% of the state territory and located in the southwest region bordering with Argentina and Uruguay, followed by the Parana-Paraiba interior forests with 63,504.9 km² (17.76%). More towards the center, the Brazilian Araucaria moist forest covers 57,758.2 km² (16.5%) and borders the State of Santa Catarina (north), and small areas of the Brazilian Atlantic coast (4,181.4 km² (1.17%)) and the Serra do Mar coastal forests (1,678.60 km² (0.47%)) (Fig 3). The Mesopotamian savannas bordering Argentina along the Uruguay River represent a very small area (113.1 km² (0.03%)). A visual exploration of the distribution of leptospirosis rates by quintiles under the ecoregions suggests that municipalities in the higher quintiles are concentrated within the Parana-Paraiba interior forests. A summary of the distribution of these variables is shown in Table 2.

**Table 2:** Possible drivers and leptospirosis cases per municipalities (P<0.15 in the univariable and multivariable analysis and P<0.05), Rio Grande do Sul, 2008–2012.

Variables	Leptospirosis cumulative incidence	Univariable analysis		Multivariable analysis	
	No.(%)*/Median (IQR)	*P*-Value	RR (CI _95%_)	*P*-Value	RR (CI _95%_)
**Environment**					
*Types of ecoregions*					
1.Parana/Paraiba Interior forest^a^					
Minority(≤ 50%)	309 (62)*	-	-	-	-
Majority(>50.01%)	187 (38)*	<0.001	2.35 (1.49–3.71)	<0.001	2.25 (2.03–2.49)
2.Brazilian Araucaria moist forest^a^					
Minority(≤ 50%)	361 (72)*	-	-	-	-
Majority(>50.01%)	135 (28)*	<0.001	0.30 (0.22–0.42)	-	-
*Types of soils*					
3.Neossolo Litolítico^a^					
Minority (≤ 50%)	393 (79)*	-	-	-	-
Majority (>50.01%)	103 (21)*	0.02	2.13 (1.11–4.10)	0.006	1.93 (1.26–2.96)
4.Red Latossolo^a^					
Minority (≤ 50%)	406 (81)*	-	-	-	-
Majority (>50.01%)	90 (19)*	0.05	0.55 (0.30–0.94)	-	-
5.Red-Yellow Argilosolo^a^					
Minority(≤ 50%)	466 (93)*	-	-	-	-
Majority(>50.01%)	30 (7)*	0.007	0.47(0.27–0.81)	-	-
6.Precipitation of the wettest month (mm)	173.1 (114.6–203.2)	<0.001	1.00 (0.97–1.00)	-	-
7.Interaction (Slope and Altitude)	540.1 (233.3–1109)	0.002	0.99 (0.99–0.99)	-	-
**Socioeconomics**					
8.Rural population	2326 (1548–3785)	0.06	1.00 (0.99–1.00)	-	-
9.Gini index	0.54 (0.28–0.73)	<0.001	0.10 (0.08–1.35)	-	-
**Productive process**					
10. Production of Rice per Tons^b^	6.5 (0–507,778)	0.14	1.00 (1.00–1.07)	<0.001	1.003 (1.002–1.04)
11.Production of Tobacco per Tons^b^	16 (0–21,080)	<0.001	1.00 (1.00–1.00)	<0.001	1.10 (1.09–1.11)
12.Proportion of bovine per municipality^c^					
Minority(≤ 50%)	280 (56)*	-	-	-	-
Majority(>50.01%)	216 (44)*	0.002	1.00 (1.00–1.00)	-	-
13.Proportion of bovine farms per km^2^	2.37 (1.20–3.43)	<0.001	1.32 (1.13–1.55)		
14.Proportion of farm per km^2^	40.04 (28.81–59.63)	0.15	1.00 (0.98–1.01)		

^a^ Variables were dichotomized as minority (was considered when covering land or proportional were less then 50% of the municipality) majority (was considered when covering land or proportional were above 50.01% of the municipality

^b^ Estimates for rice and tobacco are presented on the increase of one unit on count cases per 10.000 tons.

^c^ Variable was dichotomized as minority (considered when the less then 50% of municipality’s properties had up to 10 animals) majority (when more then 50.1% of municipality’s properties had up to 10 animals)

A closer look at the bioclimatic variables yielded medians and ranges of the monthly temperature in Celsius degrees and precipitation of the wettest month in millimeters of 18.85°C (14.53–20.92) and 173.1 mm (114.6–203.2), respectively. The average altitude in the state is 352 meters above sea level (range: 2.61–1,169.28) with 25% of the municipalities at an altitude above 157 meters. The interaction between altitude and slope showed to be significantly related to higher number of cases of leptospirosis in the univariate analysis (Table 2).

**Fig 4:**
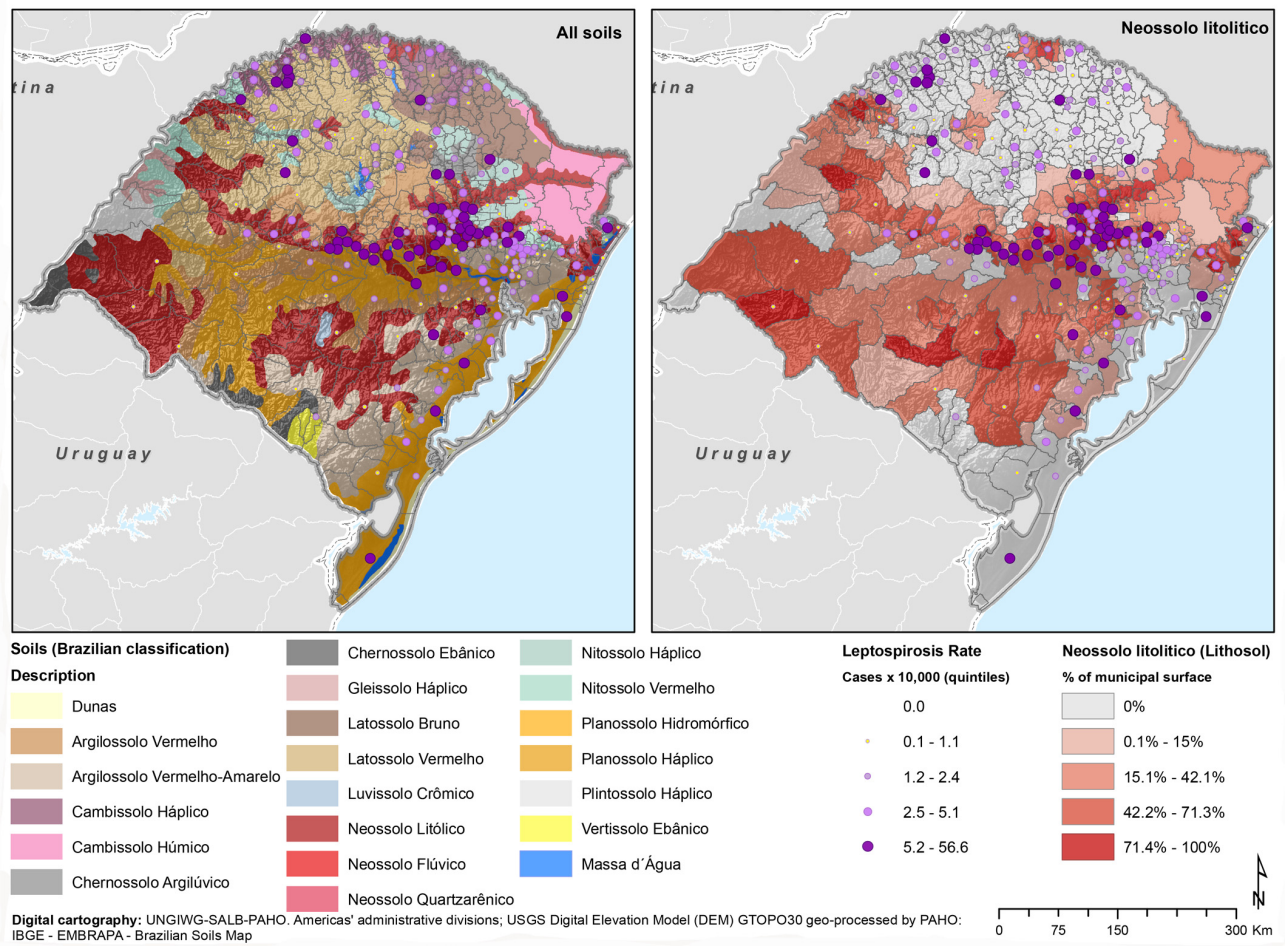
Cumulative incidence for leptospirosis type of soil and neossole type, by municipality Rio Grande do Sul, 2008–2012.

According to the Brazilian agency for soils classification [47], there are 12 types of soil in the state. The local Neossolo Litolitico (RL) soil prevails, covering 106.6 km² (23.40%), followed by Latosol Vermelho (91.29 km²) and Cambisol Haplico (52.88 km²). Neossolos Litoliticos are distributed near river valley walls surrounding the deeper and well developed Latosols soils. When analyzing the predominant types of soils individually by quintiles, the first visual impression is that a higher human leptospirosis cumulative incidence overlaps with Neossolo Litolitico (Fig 4).

#### Socioeconomic variable

The average gross domestic product (GDP) per capita in the Rio Grande do Sul State was 19,646.61 Brazilian Real (range: 7,020.39–224,004.4, median 17,121.95). The Gini index measuring inequality, in this case related to the domestic income per capita by municipality, was 0.5472 (range: 0.2841–0.73248). The illiteracy rate in the state was 6.56% (range: 0.9–19.1%, median 6.1%). The univariate analysis suggested some relation between the Gini index and the number of cases (P≤0.15), however this relationship was not observed in the final model.

#### Productive process

**Fig 5:**
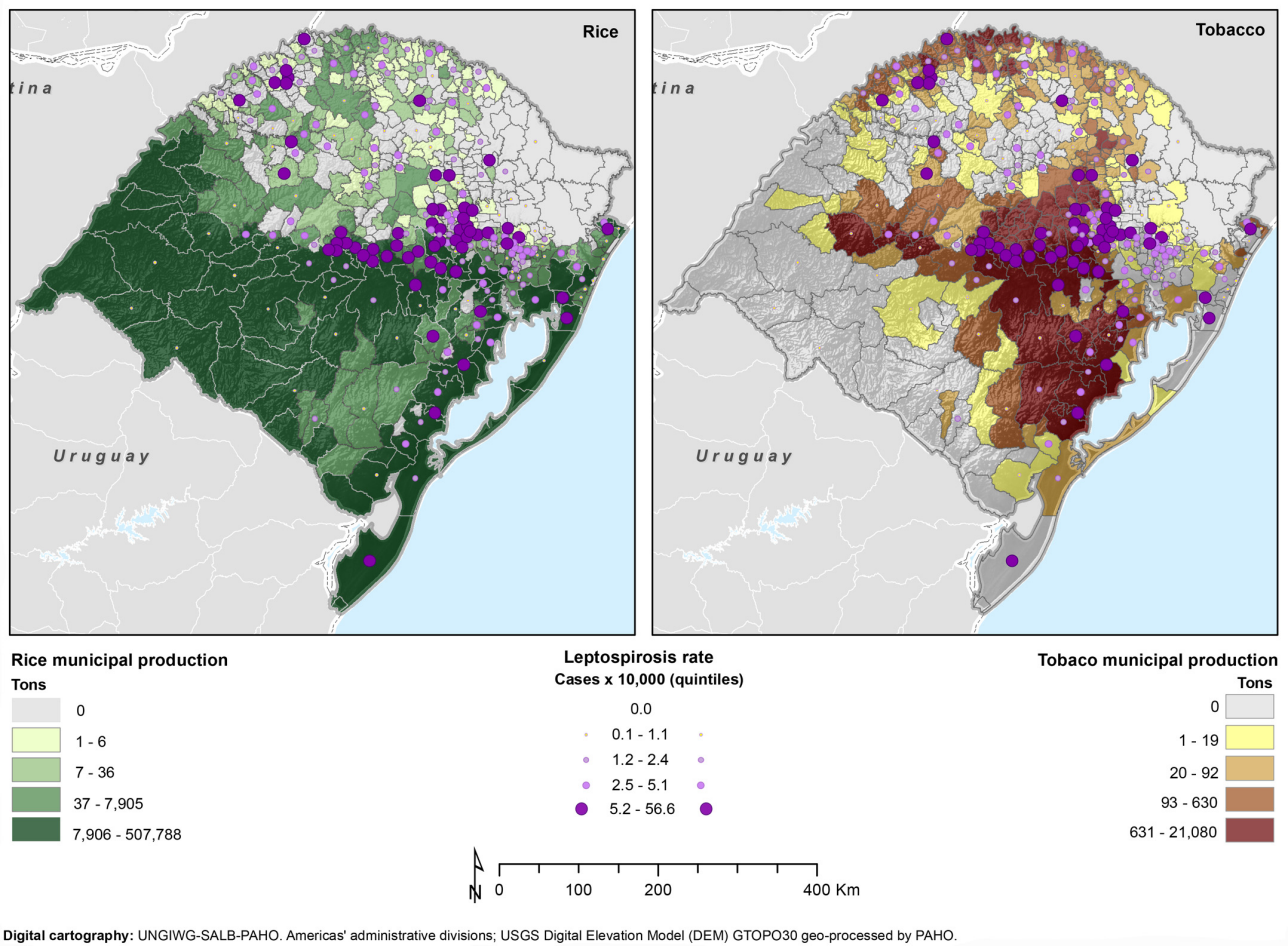
Cumulative incidence for leptospirosis, rice paddy plantation, and tobacco plantation, by municipality, Rio Grande do Sul, 2008–2012.

During 2010, the state of Rio Grande do Sul produced 6,875,077 tons of rice, with a mean of 1,3861.04 and median of 6,5 tons (range: 0–507,788). There were rice paddies in 337 of the 496 municipalities (67.94%). A large portion of the state, the entire southern half, includes municipalities in the highest quintile of rice production (Fig 5). When comparing thematic maps on rice production and leptospirosis cumulative incidence, the overlapping areas suggest a potential relationship between the two variables.

The other agricultural production analyzed was tobacco, which is grown in 319 municipalities (64.31%) with a total of 343,682 tons per year (mean: 692.91, median: 16 and range: 0–21,080). The municipalities in the highest quintile for tobacco production are located in the central and northern areas of the state, especially at lower altitudes (Fig 5). The thematic map overlapping tobacco production and leptospirosis also suggests some correlation.

**Fig 6:**
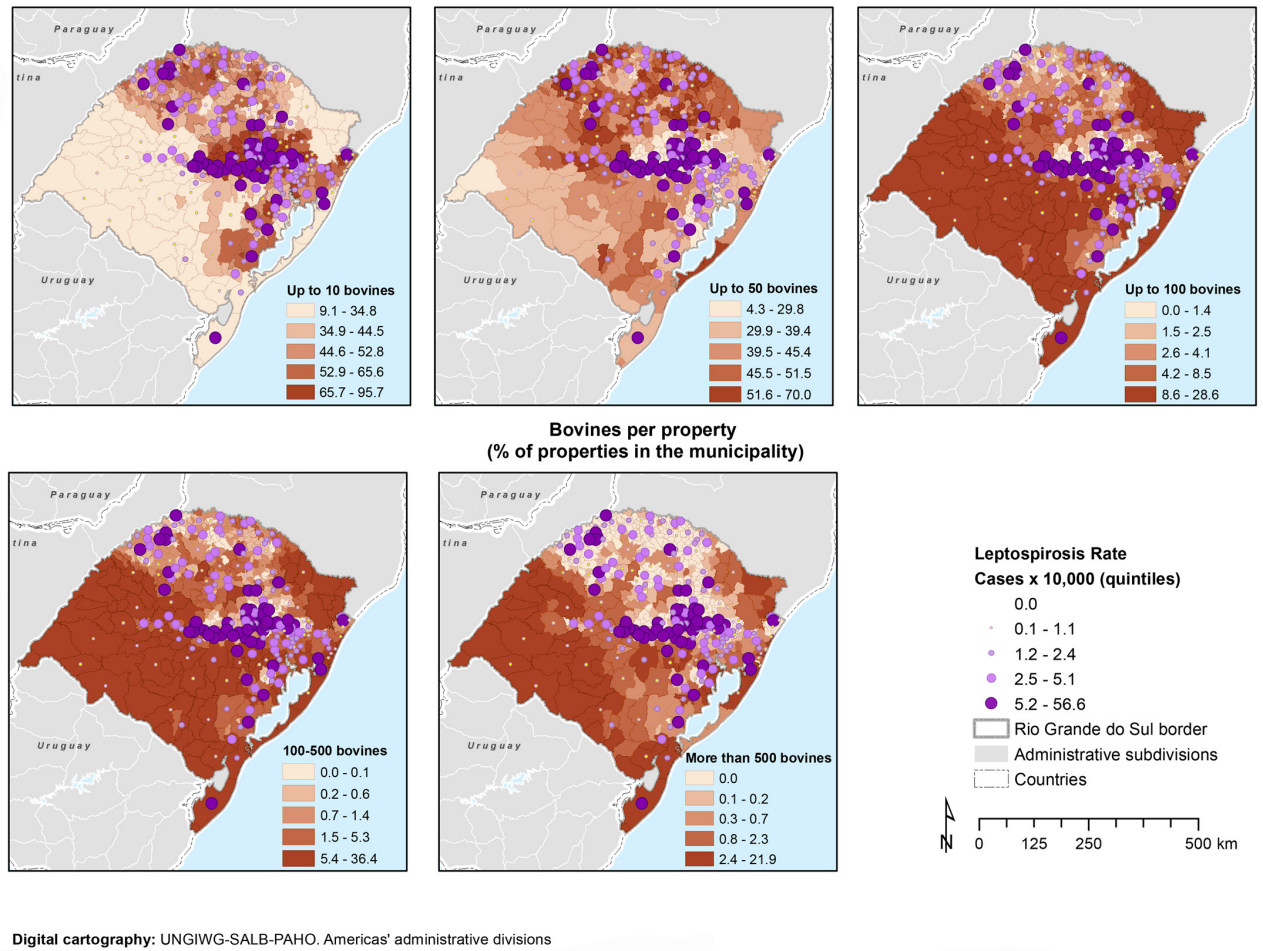
Cumulative incidence for leptospirosis and percentage of properties with up to 10 bovines, by municipality, Rio Grande do Sul, 2008–2012.

All municipalities in the state have livestock, in this case bovine, with a total of 13,541,230 animals (mean: 27,300.86, median: 9,482.5, and range: 91–633,381). A concentration of municipalities in the highest quintile of bovine per property was identified in the southwest along the border with Uruguay and Argentina (Fig 6). In the visual analysis of the number of bovine by property, a possible overlap appeared between a high number of properties with less than 10 bovines and leptospirosis cumulative incidence in the highest quintile (Fig 6). The univariate analysis suggested some relation between the number of properties with less than 10 bovines and leptospirosis cases (P≤0.15), however this relationship was not observed in the final model.

### Multivariable analysis

The negative binomial regression modeling identified fourteen variables that suggested some relation with the number of cases per municipalities in the univariate analysis (P≤0.15) (Table 2). The 12 variables that were analyzed as possible drivers (P>0.15) are presented in the S3 Supporting Information.

The final model identified four variables as significantly associated with the leptospirosis case count (P ≤ 0.05) (Table 2): the Parana/Paraiba interior forest ecoregion (RR = 2.25; IC_95%_ = 2.03–2.49; P < 0.001); Neossolo Litolítico soil (RR = 1.93; IC_95%_ = 1.26–2.96; P = 0.006); to a weaker extent, production of tobacco per 10,000 tons (RR = 1.10; IC_95%_ = 1.09–1.11; P <0.001); and finally, a borderline association with production of rice per 10,000 tons (RR = 1.003; IC_95%_ = 1.002–1.04; P <0.001). The goodness-of-fit model was tested by a deviance chi-squared test and was found not to be significant (P > 0.05).

## Discussion

Leptospirosis is an important public health problem for the state of Rio Grande do Sul, with an average of 428 cases reported annually. The actual incidence is probably much higher, particularly in vulnerable populations, because leptospirosis is commonly misdiagnosed and unreported.

The study results show that rural populations of the state have an approximately eight times higher risk of contracting leptospirosis than their urban counterparts, even though the number of reported cases was high in both areas. Rural cases are concentrated mostly in two zones, characterized by higher tobacco production (central state) and higher rice production (south). This result suggests that preventive strategies in these regions require strong collaboration between the Health and the Agriculture sectors. Preventing cases among rural workers will be the main goal for these areas.

In order to contribute to the One Health discussion and support possible intersectoral collaboration, suggestions for components of a leptospirosis plan and activities in the state of Rio Grande do Sul are presented in the S6 Supporting Information; using both the working definition of One Health and the results of this study as a basic scenario [9].

The highest concentration of urban cases was found in the metropolitan areas of cities with low altitude, including the state capital, which also suffer from frequent flooding. At the center of the state, high rate areas were found within the Atlantic East Coast watershed, which receive water from the *Jacui*, *Taquari*, *Cai*, *Sinos* and others rivers as mayor tributaries, all well known for their periodic flooding [48]. This suggests that leptospirosis prevention should also be considered in natural disaster plans and that the main goals for these areas should be to reduce the number of severe cases and save lives during an outbreak.

Other possible drivers for leptospirosis identified in the final regression model were the Parana-Paraiba ecoregion, Neossolo Litolítico soil, and the production of tobacco. The Parana/Paraiba interior forest ecoregion showed an RR of 2.25 when compared to other ecoregions. This ecoregion is part of the Atlantic semi-deciduous forest biome that covers central and northern areas of the state and extends over numerous areas of Brazil and neighboring countries. It ranges from river plains to middle-level highlands. The climate is subtropical [49] so humidity may act as an important factor in the leptospirosis bacteria survival. The Parana-Paraiba ecoregion includes a diversity of soil types that vary from very fertile to impoverished. Neossolo Litolítico, a very young and not yet structured type of soil, is one of those that correlate geographically with high rates of human leptospirosis. Its RR is 1.93 when compared with municipalities with other predominant soil type. It has been suggested that its pH [50] and/or its low drainage capacity [51] may favor the bacteria survival.

With each additional 10.000 tons of tobacco produced by the municipality, there was an increased RR in the leptospirosis cases of 1.10. There is spatial overlapping of municipalities with high production of tobacco and higher rates of the disease in the *Jacui-Tacuari* valley. In the central area, several conditions concur with higher leptospirosis cases, in particular tobacco (and rice) production in a subtropical forest environment, over slope and inclined terrain with shallow weathered soils.

We did not find information in the literature suggesting an association between tobacco plantation and leptospirosis cases. However, tobacco growth requires a pH of 5–6.5 [52], which matches the leptospirosis bacterial soil requirement of 6.2 for survival over 7 weeks in the environment [22,53]. PH survival requirements vary among serovars, and as an example *L*. *interrogans* serovar *hardjo* prefers a pH of 6.5–6.8 [54]. Further studies analyzing which serovars are circulating in humans and in animals in these high risk areas will provide additional knowledge on the cycle of leptospirosis transmission.

The agro-industrial production and export of tobacco are concentrated in three states in the south of Brazil. Rio Grande do Sul and the neighboring state of Santa Catarina are responsible for 80% of the production. Brazilian tobacco is grown by approximately 186,000 farming families in properties with an average area of 16 hectares [55]. Every step in the plantation of tobacco in Southern Brazil is carried out manually, mostly by family members from several generations of small farmers descended from Europeans immigrants [55]. Most of the farms have a small number of bovines, mostly dairy cows, although swine are also very frequent [37]. In this region, the most common feed for livestock is corn, mostly stocked on the farm where it attracts rodents. Insects and rodents are responsible for an estimated loss of 15% of all corn storage on small farm barns [47]. This region also includes large areas of preserved forests, which probably increases contact with wild rodents. Further studies about the presence of rodents and programs for rodent control would provide important information on their true impact.

In the final model, the association with rice production was weak (around 1 but statically significant), but there was some spatial overlapping of municipalities with high leptospirosis cumulative incidence and rice plantations in the Lagoa dos Patos shoreline. The state of Rio Grande do Sul produces 63% of all rice in Brazil [56]. The association between rice paddy workers and leptospirosis has already been described in the literature [6,57]. Working conditions in rice fields have been studied in Peru and results highlight occupational hazards linked to leptospirosis infection, such as long period of exposure to water, lack of use of any personal protection equipment, and presence of skin wounds [57]. It is very important to raise awareness about the risk of this disease in critical areas and promote personal protection equipment (PPE).

In the state of Rio Grande do Sul, where temperatures exceed 24°Celsius (75°Fahrenheit) for half of the year, educational campaigns for the adequate use of PPE (such as boots) among workers are unlikely to be effective, especially during long shifts. The most important prevention tool for leptospirosis in high risk areas and especially under these conditions is vaccination, however, a human vaccine is not available in the majority of affected countries.

In the central areas of the state, smaller livestock producers (less than 10 bovines by property) are predominant [37], but this variable only presented relevance in the univariate analysis. Similarly, the proportion of bovine farms per km^2^ has shown some relation with the number of leptospirosis cases. The proximity with animals such as dogs increases the risk for leptospirosis [58]. A previous study found that leptospirosis titers were present in 39% of bovines in the state [59]. Further studies are required to better understand the epidemiological situation in the animal sector.

This study provides evidence of the importance of the environmental component and the One Health approach for leptospirosis. As highlighted here, where the ecoregions were suitable for specific plantations (such as tobacco in Parana-Paraiba) human occupation of these territories has led to transformations of the natural environment and increased the use of natural resources for economic purposes [60]. Further research is required, using primary data, to further explore this finding, as well as the potential relationship between the type of ecoregion and economic processes, and their relationship with the animal-human interface. For other diseases, such as rabies transmitted by vampire bats, outbreaks have been reported rapidly in the Amazon region due to some productive processes such as gold prospection [61].

Rio Grande do Sul has one of the highest GDP per capita in the country [62]. For this reason, the study includes the Gini index that reflects possible inequalities related to the distribution of income among the inhabitants of the municipalities. However, the Gini index presented relevance only in the univariate analysis and no association was found in the final model.

This results of this study provide potentially useful information for government decision-makers in the elaboration of a possible intersectoral plan. Evidence-based policy-making requires advanced social planning capacity, including the capacity to monitor and evaluate programs in the different ministries and agencies that provide services to the most vulnerable populations. Risk stratification generates evidence for decision-making and the critical areas identified by this study could be used as pilots for future local intersectoral studies. In the same way, areas identified as silent require attention from the health authorities to confirm the absence of disease in humans or the need to improve surveillance. The prediction, detection, prevention, and response to outbreaks of leptospirosis will be improved and better defined through knowledge generated by an integrated approach within the animal-human-ecosystem interface.

This study clearly highlights the importance of a multidisciplinary and intersectoral approach, which was at the root of the creation of initiatives such as the Global Leptospirosis Environmental Action Network (GLEAN). GLEAN brings together experts from different disciplines and sectors with the objective of orienting knowledge regarding the prediction, prevention, detection, and response to leptospirosis; and transforming relevant research findings into operational tools for affected communities and countries, as well as for organizations [63].

Although the use of aggregated data by municipality is a possible limitation of this study, the geographical approach helped to gather, unify and shape all factors (environmental, socioeconomic, and health) into the same area and detect spatial patterns. Ecological studies are an inexpensive way to use secondary available data; however they are commonly associated with the ecological fallacy [21]. Another limitation possibly resides in the large number of municipalities with small populations (under 10,000 people). However, the possible effect of this data characteristic was considered in the statistical analysis through the use of negative binomial regression, by testing the effects using strata with and without small population. Also, no significant difference was found by analyzing the same approach using only municipalities with at least one positive case.

This study contributes to the basic knowledge on leptospirosis distribution and drivers in the state of Rio Grande do Sul and points to the direction of tool-readiness for the disease. It also encourages a holistic approach for the disease, taking into account human, animal, and ecosystem interactions and supporting research in the development and validation of a rapid diagnostic test for humans and animals, and eventually production of a vaccine that is still desperately needed.

## Supporting Information

S1 Supporting Information Sources of information. (DOCX)

S2 Supporting Information Map of hydrology and leptospirosis cumulative incidence. (DOCX)

S3 Supporting Information Table of possible drivers and leptospirosis rates (P>0.15). (DOCX)

S4 Supporting Information Details about modeling comparison and verifications. (DOCX)

S5 Supporting Information Map of critical areas for leptospirosis. (DOCX)

S6 Supporting Information Components and activities for a leptospirosis plan in the state of Rio Grande do Sul. (DOCX)

S7 Supporting Information Abstract in Spanish. (DOCX)

S8 Supporting Information Abstract in Portuguese. (DOCX)

S9 Supporting Information STROBE Checklist. (DOC)
