# Supplementary material for: Leptospirosis in Rio Grande do Sul, Brazil: An Ecosystem Approach in the Animal-Human Interface
Source: PLoS Negl Trop Dis. 2015 Nov 12;9(11):e0004095. doi: 10.1371/journal.pntd.0004095 (PMC4643048; doi:10.1371/journal.pntd.0004095)
Supplement: S1 Supporting Information — (DOCX) [file pntd.0004095.s001.docx]

**Supporting Information S1**

**Table 1.** Selected variables and sources of information used to create a database by municipality.

| **Variables taken from original sources** | **Sources** |
| --- | --- |
| Name of the Municipality | IBGE[1] |
| Municipality total area | IBGE[1] |
| Demographic density | IBGE[1] |
| Total rural population | IBGE[1] |
| Code of Second National Sublevel  Population by Municipality 2010  Relative Urban Population 2010  Total Area in (Km²)  Number of Cases of Leptospirosis (2008-2012)  GINI Index by Municipality 2010  PIB per capita by Municipality 2010  Illiteracy Rate by Municipality 2010  Production of Rice and Tobacco per Tons by Municipality 2010 | IBGE[1]  IBGE [1]  IBGE [1]  IBGE[1]  SINAN[2]  IBGE/ DATASUS[3]  IBGE/ DATASUS[4]  IBGE/ DATASUS[5]  IBGE/ SIDRA[6] |
| Number of bovines | SEAPA[7] |
| Percentage of bovine per property | SEAPA [7] |
| Number of Bubaline | SEAPA [7] |

| **Variables created from original sources** | **Sources** |
| --- | --- |
| Cumulative incidence rate by 10,000 population | SINAN/IBGE[8] |
| Altitude (in meters above sea level) | USGS HYDRO1K [9] |
| Slope of the land | USGS HYDRO1K [9] |
| Type of Soil | AMBDATA[10] |
| Ecoregions | FAO Geonetwork/ WWF[11] |
| Annual mean temperature | BIOCLIM [12] |
| Precipitation of the wettest month (mm) | BIOCLIM [12] |
| Slope of the land | USGS HYDRO1K [9] |
| Drainage | USGS HYDRO1K [9] |
| Basins | USGS HYDRO1K[9,11,13] FAO HydroSHEDS rivers SEMA- SEGPLAN RS/DEPLAN |

**Sources:**

1. IBGE - Fundação Instituto Brasileiro de Estatística. 2014. [cited 2015 Jan 15]. Brasilia, Brasil: Ministério da Saúde. c 2014. Available from: http://downloads.ibge.gov.br/downloads_estatisticas.htm.
2. Sistema de informação de agravos de notificação [Internet]. Brasilia, Brasil: Ministério da Saúde. c 2014 - [cited 2015 Feb 24]. Available from: http://dtr2004.saude.gov.br/sinanweb/ .
3. Informações de saúde. Datasus [Internet]. Brasilia, Brazil: Ministério da Saúde. c 2010 - [cited 2015 Feb 24]. Available from: <http://tabnet.datasus.gov.br/cgi/ibge/censo/cnv/ginirs.def> .
4. Saraiva A. IBGE: maior PIB per capita entre arranjos populacionais está no RS. Valor Econômico [Internet]. Rio de Janeiro, Brasil: Alessandra Saraiva. 2015 Mar 25 - [cited 2015 Apr 2]. Available from: <http://www.valor.com.br/brasil/3974750/ibge-maior-pib-capita-entre-arranjos-populacionais-esta-no-rs>. Portuguese.
5. Informações de saúde-Analfabetismo 2010. Datasus [Internet]. Brasilia, Brasil: Ministério da Saúde. c 2010 - [cited 2015 Feb 24]. Available from: http://tabnet.datasus.gov.br/cgi/tabcgi.exe?ibge/censo/cnv/alfrs.def.
6. IBGE- SIDRA- Banco de dados agregados. [cited 17 March 2015]. In: IBGE [Internet]. Brasília, Brasil- . [about 2 screens]. Available: <http://www.sidra.ibge.gov.br/bda/tabela/listabl.asp?c=1612&z=t&o=11>.
7. Secretaria da Agricultura, Pecuária e Agronegócio do Rio Grande do Sul (SEAPA-RS). Departamento e Defesa Agropecuária. Relatório Anual de Atividades – 2012. 163 p.
8. Sistema de informação de agravos de notificação [Internet]. Brasilia, Brasil: Ministério da Saúde. c 2014 - [cited 2015 Feb 24]. Available from: http://dtr2004.saude.gov.br/sinanweb/
9. Science for a changing world (USGS) [Internet] [cited 2015 Mar 14]. Available from : <https://lta.cr.usgs.gov/HYDRO1K>.
10. Variáveis ambientais para modelagem de distribuições de espécie-AMBDATA- [cited 5 March 2015]. In: Divisão de Processamento de Imagens [Internet]. Brasil [about 2 screens]. Available: http://www.dpi.inpe.br/Ambdata/referencias.php.
11. FAO-GeoNetwork-geo-spatial – World Wild Fund Global Ecoregions Map[Internet]. [cited 2014 Dec 10]. Available from: <http://www.fao.org/geonetwork/srv/en/main.home>.
12. WorldClim – Global Cimate data. [Internet]. California, United States of America. c 2015 - [cited 2015 Feb 10]. Available from: http://www.worldclim.org/bioclim.
13. Secretaria de Ambiente e Desenvolvimento Sustentável-SEMA. 2010 [Internet]. Porto Alegre, Brasil. c 2010 - [cited 2015 Jun 2]. Available from: http://www.sema.rs.gov.br/.
