# Supplementary material for: Leptospirosis in Rio Grande do Sul, Brazil: An Ecosystem Approach in the Animal-Human Interface
Source: PLoS Negl Trop Dis. 2015 Nov 12;9(11):e0004095. doi: 10.1371/journal.pntd.0004095 (PMC4643048; doi:10.1371/journal.pntd.0004095)
Supplement: S3 Supporting Information — (DOCX) [file pntd.0004095.s003.docx]

**Supporting Information S3**

Possible drivers and leptospirosis rates (P>0.15), Rio Grande do Sul, 2008-2012

| Variables | Leptospirosis rates | | Univariate analysis |
| --- | --- | --- | --- |
|  | No.(%)*/Median (IQR) | *P*-Value | RR (CI _95%_) |
| Demographic |  |  |  |
| Density | 23.32 (13.68-42.74) | 0.20 | 0.99 (0.99-1.00) |
| Environment |  |  |  |
| Annual mean temperature | 18.8 (14.53-20.92) | 0.47 | 1.01 (0.30-1.04) |
| Total Area in (Km²) | 237.20 (124.7-506) | 0.97 | 1.00 (0.99-1.00) |
| Slope of the land | 1.48 (0.99-2.46) | 0.72 | 1.03 (0.83-1.28) |
| Drainage | 5.74 (5.06-6.19) | 0.17 | 1.04 (0.99-1.32) |
| *Types of soils* |  |  |  |
| Planosolo Háplico^a^ |  |  |  |
| Minority(≤ 50%) | 470 (94)* | - | - |
| Majority(>50.01%) | 26 (6)* | 0.45 | 1.23 (0.71-2.10) |
| Red Arginosolo^a^ |  |  |  |
| Minority (≤ 50%) | 456 (91)* | - | - |
| Majority (>50.01%) | 40 (9)* | 0.18 | 0.79(0.57-1.11) |
| *Types of ecoregions* |  |  |  |
| Uruguayan savanas^a^ |  |  |  |
| Minority (≤ 50%) | 342 (68)* | - | - |
| Majority(>50.01%) | 154 (32)* | 0.27 | 0.77 (0.48-1.22) |
| Socioeconomics |  |  |  |
| PIB per capita | 17120 (13480-22900) | 0.72 | 1.00 (0.99-1.00) |
| Illiteracy rate | 6.10 (3.90-8.52) | 0.90 | 1.00 (0.94-1.06) |
| Productive process |  |  |  |
| Total No. of bovine | 9482 (4874-20480) | 0.42 | 0.99 (0.99-1.00) |
| Total No. of bubaline | 2 (0-47.75)* | 0.18 | 0.99 (0.99-1.00) |
