# Supplementary material for: Leptospirosis in Rio Grande do Sul, Brazil: An Ecosystem Approach in the Animal-Human Interface
Source: PLoS Negl Trop Dis. 2015 Nov 12;9(11):e0004095. doi: 10.1371/journal.pntd.0004095 (PMC4643048; doi:10.1371/journal.pntd.0004095)
Supplement: S4 Supporting Information — (DOCX) [file pntd.0004095.s004.docx]

**Supporting Information S4**

**Details about modeling comparison and verifications**

The outcome variable tested was the Leptospirosis count cases per municipalities where there were 53% had zero cases. To better address this outcome we verified and did compared the recommended approach for such data. The approach by Poisson regression is one of the models from general linear models for describing count outcomes or proportion (1). Eventually in Poisson the variances are much larger than the means, whereas Poisson distributions have identical mean and variance (2), the phenomenon of the data having greater variability than expected for a general linear model is called over-dispersion. A regular cause of over-dispersion is heterogeneity among subjects (1). A Negative Binomial model, is another model from general linear models as an alternative to the Poisson model, and is a solution to account for over-dispersion due to unobserved heterogeneity (3). Alternatively, if the non-zero observation part does not follow the Poisson model then the zero-inflated Negative binomial is used by considering count process as a negative binomial distribution (4). The zero-inflated Negative binomial model provides the possibility that account for the over-dispersion due to both types of excess zeros and unobserved heterogeneity (4). The models: Poisson regression versus negative binomial and zero-inflated Negative binomial, were compared using the Vuong test and likelihood ratio test. To compare performance of the models, Akaike Information Criterion (AIC) and Bayesian Information Criterion (BIC) were used. Significant P-value of 0.05 was used.

| Combinations | AIC | P-value | BIC | P-Value |
| --- | --- | --- | --- | --- |
| 1. Negative binomial vs. (2)Zero-inflated Negative Binomial | 0.68 | 0.68 | 1.56 | 0.05 |
| 1. Negative binomial vs. (2)Poisson regression | 3.33 | <0.001 | 3.33 | <0.001 |
| 1. Zero-inflated Negative Binomial vs. (2)Poisson regression | 3.34 | <0.001 | 3.30 | <0.001 |

A large, positive test statistic (AIC and/or BIC) provides evidence of the superiority of model 1 over model 2, while a large, negative test statistic is evidence of the superiority of model 2 over model 1. Under the null that the models are indistinguishable, the test statistic is asymptotically distributed standard normal. One can conclude that using this approach the Negative binomial is a little superior to Zero-inflated negative binomial and superior to Poisson regression, that support our chose for the use of Negative binomial on this data.

References:

1. Agresti A. An Introduction to Categorical Data Analysis; John Wiley & Sons; INC, publication; 2007.
2. Baghban AA, Zayeri F, Alavian SM, Mohsen Vahedi M. Statistical count models for prognosis the risk factors of hepatitis C. Gastroenterol Hepatol Bed Bench. 2013. 6(1):41–47.
3. Dwivedi AK, Dwivedi SN, Deo S, Shukla R, Kopras E. Population of Iran Statistical models for predicting number of involved nodes in breast cancer patients. Health. 2010;2:641–51.
4. Lambert D. Population of Iran Zero-Inflated Poisson Regression, with an Application to Defects in Manufacturing. Thecnometrics. 1992; 34:1-14.
