# Supplementary material for: Leptospirosis in Rio Grande do Sul, Brazil: An Ecosystem Approach in the Animal-Human Interface
Source: PLoS Negl Trop Dis. 2015 Nov 12;9(11):e0004095. doi: 10.1371/journal.pntd.0004095 (PMC4643048; doi:10.1371/journal.pntd.0004095)
Supplement: S5 Supporting Information — (DOCX) [file pntd.0004095.s005.docx]

**Supporting Information S5**

Critical areas for leptospirosis, by municipality, Rio Grande do Sul, 2008-2012
