# Supplementary material for: Leptospirosis in Rio Grande do Sul, Brazil: An Ecosystem Approach in the Animal-Human Interface
Source: PLoS Negl Trop Dis. 2015 Nov 12;9(11):e0004095. doi: 10.1371/journal.pntd.0004095 (PMC4643048; doi:10.1371/journal.pntd.0004095)
Supplement: S6 Supporting Information — (DOCX) [file pntd.0004095.s006.docx]

**Supporting Information S6**

Components and activities for a leptospirosis plan in the state of Rio Grande do Sul based on the One Health working definition*

| **Components** | | |
| --- | --- | --- |
| **Human** | **Animal** | **Ecosystem** |
| More than 400 human cases year officially reported per year, including around 10% of fatalities; sick people will need health care, some hospitalization in high complexity care such as hemodialysis. The patient and their families will be affected in their everyday life by the disease, and the most vulnerable families such as farmers could be economically affected. | Different domestic and wild species carrier and spill over the bacterium in the environment through urine, transmitting the disease to others animals and people. Clinical cases in domestic animals generate economic losses (abortion and decline in productivity). A few surveys have been done in domestic animals in the state of Rio Grande do Sul. Wild rodents are abundant in the state, among them the *Myocastor coypus* (“ratão-do-banhado”), which is known for carrying and shedding leptospires in fresh water in Europe, not yet studied in the south of Brazil [1,2,3]. | Areas with environmental conditions favorable for outbreaks of leptospirosis such as floods (metropolitan region of Porto Alegre and the Taquari and Cai rivers basis). Types of soils with a pH and structure that allow for longer survival of the bacterium such Neossole Litolitico in the Center area of the state. Certain use of land such as rice paddy plantation, very common in the state Rio Grande do Sul. |
| **Possible activities** | | |
| -Develop driver’s analysis and evidence based studies integrating data about leptospirosis in humans and animals, environmental and socioeconomic variables to forecast risk areas.  - Improve awareness and prevention activities about leptospirosis risk in rice and tobacco plantations areas, in collaboration with the Health and Agriculture sectors, as well as with the involvement of civil societies ~~as well~~, such as farmer associations.  - Include leptospirosis in Civil Defense Flood Plans and other disaster relief strategies, as well as identify flood-prone areas and the period of greater precipitation to predict possible outbreaks [4,5,6].  -Elaborate an intersectoral plan, including share information and coordinate actions of prevention and control leptospirosis, as well as joint investigation of possible outbreaks [4, 7].  -Elaborate intersectoral guidelines and joint training multidisciplinary teams, at the different levels; and also awareness/education materials [7,8]. | | |

*Please refer to the working definition of One Health on reference 9.

References:

1. Waitkins SA, Wanyangu S, Palmer M. J Hyg (Lond).The coypu as a rodent reservoir of leptospira infection in Great Britain. 1985 Oct; 95(2):409-17.
2. Michel V, Ruvoen-Clouet N, Menard A, Sonrier C, Fillonneau C, Rakotovao F, Ganière JP, André-Fontaine G. Role of the coypu (Myocastor coypus) in the epidemiology of leptospirosis in domestic animals and humans in France. Eur J Epidemiol. 2001;17(2):111-21.
3. Aviat F, Blanchard B, Michel V, Blanchet B, Branger C, Hars J, Mansotte F, Brasme L, De Champs C, Bolut P, Mondot P, Faliu J, Rochereau S, Kodjo A, Andre-Fontaine G. Leptospira exposure in the human environment in France: A survey in feral rodents and in fresh water. Comp Immunol Microbiol Infect Dis. 2009; 32(6):463-76
4. Schneider MC, Nájera P, Aldighieri S, Bacallao J, Soto A, Marquiño W et al. Leptospirosis outbreaks in Nicaragua: Identifying critical areas and exploring drivers for evidence-based planning. Int J Environ Res Public Health. 2012; 9(11):3883-3910.
5. Weinberger D, Baroux N, Grangeon JP, Ko AI, Gorant C. El Niño Southern oscillation and leptospirosis outbreaks in New Caledonia. PLoS Negl Trop Dis. 2014 Apr 17; 8(4):e2798.
6. Barcellos C, Lammerhirt CB, de Almeida MA, dos Santos E. Distribuição espacial da leptospirose no Rio Grande do Sul, Brasil: recuperando a ecologia dos estudos ecológicos. Cad Saude Publica. 2003 Sep-Oct; 19(5):1283-92. Portuguese.
7. PAHO/WHO PROGRAM: Leptospirosis. 2013 Sep 1 [cited 17 March 2015]. In: PAHO/WHO web [Internet]. Washington DC: WHO-[about 1 screen]. Available from: http://www.paho.org/hq/index.php?option=com_topics&view=article&id=346&Itemid=40934.
8. Know Lepto. [cited 17 March 2015]. In: FLAG – Farmer Leptospirosis Action Group web [Internet]. New Zealand-[about 2 screens]. Available from: http://www.leptospirosis.org.nz.
9. Rabinowitz PM, Kock R, Kachani M, Kunkel R, Thomas J, Gilbert J et al. Toward proof of concept of One Health approach to disease prediction and control. Emerg Infect Dis. 2013 Dec; 19(12). doi: 10.3201/eid1912.130265.
