# Supplementary material for: Leptospirosis in Rio Grande do Sul, Brazil: An Ecosystem Approach in the Animal-Human Interface
Source: PLoS Negl Trop Dis. 2015 Nov 12;9(11):e0004095. doi: 10.1371/journal.pntd.0004095 (PMC4643048; doi:10.1371/journal.pntd.0004095)
Supplement: S7 Supporting Information — (DOCX) [file pntd.0004095.s007.docx]

**Supporting Information S7: Alternative language abstract – Spanish**

**Leptospirosis en Rio Grande do Sul, Brasil: Un enfoque eco sistémico en la interface animal-humana**

***Antecedentes:*** Leptospirosis es una enfermedad desatendida propensa a desarrollar epidemias la cual afecta humanos y animales principalmente en poblaciones vulnerables. El enfoque de “Una sola salud” es una estrategia recomendada para comprender mejor los factores conductores de la enfermedad y el planear para su prevención y control. El objetivo de este estudio fue analizar la distribución de los casos humanos de leptospirosis en el Estado de Rio Grande do Sul, Brasil y también explorar los posibles factores conductores. Adicionalmente, se busca aportar evidencia para las intervenciones y para identificar las hipótesis para nuevos estudios en la interface hombre-animal-ecosistema.

***Metodología y Resultados:*** El riesgo de infección humana fue descrita en relación a factores ambientales, socioeconómicos, y de ganadería. El estudio uso datos agregados por municipalidad (todas 496). Los datos fueron obtenidos de fuentes secundarias y abiertas al público. Mapas temáticos fueron construidos y un análisis univariado fue realizado para todas las variables. Una regresión negativa binomial fue utilizada en el análisis estadístico multivariado con casos de leptospirosis. Un promedio de 428 casos humanos de leptospirosis fue reportado anualmente entre los años 2008 a 2012 en el estado. La tasa de incidencia en la población rural fue ocho veces más alta de la población urbana. Los variables asociadas significativamente con casos de leptospirosis en el modelo final fueron: Ecoregion Parana-Paraiba (RR: 2.25; CI _95%_: 2.03-2.49); suelo Neossolo Litolitico (RR: 1.93; CI_95%_: 1.26-2.96); relación más débil con la producción de tabaco (RR: 1.10; CI_95%_: 1.09-1.11) y arroz (RR: 1.003; CI_95%_: 1.002-1.04).

***Conclusión:*** Los casos urbanos fueron más concentrados en la capital y los casos rurales en eco-regiones especificas. Los factores conductores principales identificados en este estudio fueron relacionados al ambiente y a los procesos de producción que seguirán siendo presentes en el estado Este estudio contribuye para el conocimiento básico sobre la distribución de la leptospirosis y los factores conductores en el estado, e incita un enfoque holístico para la enfermedad.
