# Supplementary material for: Leptospirosis in Rio Grande do Sul, Brazil: An Ecosystem Approach in the Animal-Human Interface
Source: PLoS Negl Trop Dis. 2015 Nov 12;9(11):e0004095. doi: 10.1371/journal.pntd.0004095 (PMC4643048; doi:10.1371/journal.pntd.0004095)
Supplement: S8 Supporting Information — (DOCX) [file pntd.0004095.s008.docx]

**Supporting Information S8: Alternative language abstract – Portuguese**

**Leptospirosis no Rio Grande do Sul, Brasil: Uma abordagem por ecossistema na interface animal-humana**

***Antecedentes:*** A leptospirose é uma doença desatendida propensa a desenvolver epidemias que afeta seres humanos e animais, principalmente em populações vulneráveis. O enfoque de "Uma Saúde" é uma estratégia recomendada para compreender melhor os fatores condutores da doença e planejar sua prevenção e controle. O objetivo deste estudo foi analisar a distribuição dos casos humanos de leptospirose no Estado de Rio Grande do Sul, Brasil e também explorar os possíveis fatores condutores. Além disso, busca-se contribuir com evidência para dar apoio a intervenções e para identificar hipóteses para novos estudos na interface homem-animal-ecossistema.

***Metodologia e Resultados:*** O risco de infecção humana foi descrito em relação a fatores ambientais, socioeconômicos, e de agropecuária. O estudo usou dados agregados por município (todos 496). Os dados foram obtidos de fontes secundárias e abertas ao público. Mapas temáticos foram construídos e uma análise univariada foi realizada para todas as variáveis. Uma regressão negativa binomial foi utilizada na análise estatística multivariada com casos de leptospirose. Uma media de 428 casos humanos de leptospirose foram registrados anualmente entre os anos 2008 a 2012 no estado. A incidência acumulada na população rural foi oito vezes mais alta da população urbana. As variáveis associadas significativamente com casos de leptospirose no modelo final foram: Ecoregião Parana-Paraiba (RR: 2.25; CI_95%_: 2.03-2.49); solo Neossolo Litolitico (RR: 1.93; CI_95%_: 1.26-2.96); relação mais débil com a produção de tabaco (RR: 1.10; CI_95%_: 1.09-1.11) e arroz (RR: 1.003; CI_95%_: 1.002-1.04).

***Conclusão:*** Os casos urbanos foram mais concentrados na capital e os casos rurais em eco-regiões específicas. Os fatores condutores principais identificados neste estudo foram relacionados ao ambiente e aos processos produtivos que continuarão estando presentes no estado. Este estudo contribui para o conhecimento básico sobre a distribuição da leptospirose e os fatores condutores no estado, e encoraja uma abordagem holística para a doença.
